# Gut microbial profiles of COVID-19 patients in Uganda

**DOI:** 10.4314/ahs.v26i1.2

**Published:** 2026-03

**Authors:** David Patrick Kateete, Christopher Lubega, Emmanuel Nasinghe, Monica Mbabazi, Ronald Galiwango, Daudi Jjingo

**Affiliations:** 1 Department of Immunology and Microbiology, School of biomedical Sciences, Makerere University, Kampala Uganda; 2 Makerere University School of Public Health; 3 African Center of Excellence in Bioinformatics and Data Intensive Sciences, College of Health Sciences, Makerere University, Kampala, Uganda; 4 The Infectious Diseases Institute, Makerere University, Kampala, Uganda; 5 Department of Computer Science, College of Computing and Information Sciences, Makerere University, Kampala, Uganda

**Keywords:** Gut microbiome, COVID-19, Metagenomics, Machine Learning, Kampala, Uganda

## Abstract

**Background:**

The role of the microbiome in COVID-19 outcomes remains an area of exploration. We comprehensively explored the gut microbiome of Ugandan COVID-19 patients and inferred potential implications.

**Methods:**

Stool and demographic data were collected from 100 COVID-19 confirmed cases at the covid isolation and treatment centers in Kampala during the first and second waves of the pandemic in Uganda (2020 and 2021, respectively). 16S rRNA sequencing was performed on the DNA extracted from stool, followed by bioinformatics analysis. Machine-learning techniques were used to determine microbes that were associated with disease severity.

**Results:**

We observed differences in microbial composition between COVID-19 patients and healthy controls. Pathogenic bacteria such as Klebsiella oxytoca, Salmonella enterica and Serratia marcescens had an increased presence in COVID-19 disease states, especially severe cases. Additionally, there was an increase in opportunistic pathogens like Enterococcus species, along with a decrease in beneficial microbes, such as Alphaproteobacteria, when comparing mild and severe cases. Machine-learning identified age and microbes like Ruminococcaceae, Bacilli, Enterobacteriales, porphyromonadaceae and Prevotella copri as predictive of severity.

**Conclusion:**

The microbiome likely plays a role in the dynamics of SARS-CoV-2 infection in Ugandan patients. The shift in abundance of specific microbes can moderately predict severity of COVID-19 in this population.

**Clinical trial number:**

Not applicable.

## Background

COVID-19 is a contagious respiratory disease that spread rapidly around the world resulting in a pandemic that paralyzed global health and economies of many countries. Evidence suggests alterations in the composition of the microbiota/microbiome (i.e., a collection of living microorganisms found in a defined environment, e.g., on the skin or oral, respiratory and gut mucosa) following infection with severe acute respiratory syndrome coronavirus 2 (SARS-CoV-2), the causative agent of COVID-19^1^-^4^. Such perturbations may predispose worse outcomes including increased disease severity and mortality, as well as predisposing patients to co-infections^5^,^6^. There have been reports on coinfection in COVID-19 patients, and thus far, the results vary among different populations^5^. Bacteria were found to be the dominant form of co-infection in COVID-19 patients, with Streptococcus pneumoniae being the most common pathogen followed by Klebsiella pneumoniae^6^-^8^. While distinguishing colonization from infection presents a challenge particularly in the context of COVID-19^9^-^11^, the microbiome, as well as prevalence and characteristics of bacterial coinfection in COVID-19 patients in the low-income countries, is still not well understood. Moreover, the gut microbiome composition of the Ugandan population could be uniquely influenced by high genetic diversity given several diverse ethno-linguistic groups in Uganda^12^, widespread irrational antibiotic use^13^, diet and endemic diseases such as Malaria.

Bacterial diversity analysis through 16S rRNA gene sequencing is a common method for identifying and comparing bacteria in a microbiome; although its focus is restricted to bacteria, it offers a rapid approach for gaining insights into the composition/function of the microbiota/microbiome, in addition to increasing pathogen diagnostic rates when compared to traditional workflows which largely rely on pathogen-specific tests^14^-^16^. Furthermore, artificial intelligence (AI) / machine learning (ML) approaches are increasingly being used in bacterial diversity analysis to explore the host-microbiome associations and their relation to development and progression of disease. This is due to AI/ML's superiority in dealing with high dimensional data compared to traditional computational approaches^17^,^19^. In this study, we performed ML-based analysis of 16S rRNA sequencing data from COVID-19 patients from Uganda, obtained during the initial waves of the pandemic in the country in the period between 2020 to 2022.

## Methods

### Study setting, participants and samples

This study was conducted on covid samples collected during the first two waves of the pandemic in Uganda i.e., 2020-2022. Uganda reported her first case of COVID-19 on the 21st March of 2020 and following a brief period of few cases, a large wave of cases (i.e., Uganda's first covid wave) swept through the country peaking in the second half of 2020. The second COVID-19 wave was attributed to the Delta strain; it started in early 2021 and peaked in June and continued through August 2021^20^. The Delta wave grew more rapidly than the first wave and it was associated with many fatalities in Uganda and beyond.

For this study, stool and demographic data were collected from 100 randomly selected COVID-19 patients admitted at Mulago National Referral Hospital and other treatment centres around Kampala. The diagnosis of COVID-19 was made by the on-duty clinician based on the Ministry of Health guidelines (i.e., clinical symptoms and/or molecular detection of SARS-CoV-2 in the nasopharyngeal sample). Samples of faeces and demographic data were also collected from five household healthy contacts of the cases as a comparator group. Samples (faeces) were collected using sterile plastic containers and stored at the Integrated Biorepository of H3Africa Uganda^21^. The participants were categorized into three groups: “mild COVID-19 cases” (n = 42), “severe COVID-19 cases” (n = 58), and controls (n = 5). The household healthy contacts had no visible sign of illness, tested negative on PCR for SARS-CoV-2 detection and did not report use of antibiotics at least 3 months prior to enrolment.

### 16S rRNA gene sequencing and diversity analysis

Microbial DNA was extracted from the stool samples using the TIANamp Micro DNA Kit (DP316, TIANGEN BIOTECH) according to the manufacturer's guidelines. Briefly, following DNA extraction, libraries were prepared by DNA fragmentation and addition of adapters and loaded onto a flow cell where they underwent bridge amplification to form clusters. Each cluster was then sequenced on the illumina MiSeq platfor^22^ from both ends i.e., paired-end sequencing to obtain the raw reads (approx. 250 base pair [bp] for each forward and reverse read). We used commercial primers targeting the V3-V4 region of the 16S rRNA gene. Sequencing was performed at the Makerere University Genomics/Molecular Diagnostics Laboratory at Mulago hill. The paired-end reads were demultiplexed based on their unique indices. Taxonomic classification was done using Kraken 2, which uses a k-mer based approach to compare fragments of the sequences to a user-specified database, enabling classification of the microbial composition in each sample^23^. For this analysis the Greengenes database, a well-curated reference database specializing in 16S rRNA sequences, was used^24^. Subsequent analyses were done using R Vegan^25^, Phyloseq^26^ and DESeq2^27^ packages. All plots and statistical analyses were conducted with R v4.3.1^28^. The vegan package in R was used to calculate the Shannon index for alpha diversity and Bray-Curtis dissimilarities for beta diversity. The differences in alpha diversity among or between severe and mild cases were statistically assessed using Kruskal-Wallis test. The statistical differences between the beta-diversity indices were computed using permutational multivariate analysis of variance (PERMANOVA) with the adonis() function in the vegan package^25^. Differential abundance analysis was performed using the DESeq2 package^27^ and P-values were adjusted for multiple testing using the false-discovery rate correction.

Machine Learning: We predicted severity of COVID-19 cases using demographic characteristics and bacterial composition in each sample. Following pre-processing, the data was split into training and testing datasets. Several models were trained including Logistic Regression, Random Forests, Gradient Boosting, and Neural network followed by computation of accuracy metrics to evaluate model performance. The best performing model was used to predict clinical severity of COVID-19 from the bacterial profiles. Feature importance metrics were used to obtain taxa that are significantly associated with disease severity.

## Results

### Demographics

Stool samples from a total of 105 participants (100 cases and 5 controls) were sequenced; the paired-end DNA sequences, together with the clinical and/or demographic data were analyzed for microbial profiles. The participants were categorized as ‘mild’ COVID-19 cases (n=42), ‘severe’ COVID-19 cases (n=58), and a comparator group of 5 household healthy contacts with no COVID-19 symptoms and generally in good health. Table 1 shows the participants' demographic and clinical characteristics. Participants in the ‘severe’ group were generally older with a mean age of 45.5 years compared to a mean age of 32.6 years for participants in the ‘mild’ group. While distribution by gender was relatively balanced across groups, majority of the participants were married (55.2%). Further, majority of the ‘mild’ cases reported no comorbidities (76.2%), whereas the ‘severe’ group had a higher proportion of individuals with at least one comorbidity (43.1%), Table 1.

**Table 1:** Clinical and demographic characteristics of the participants (n=105)

	Mild cases (n=42)	Severe cases (n=58)	Healthy contacts (n=5)	Total (n=105)
**Age**				
Median [Min, Max]	28.0 [15.0, 88.0]	46.5 [0, 85.0]	22.0 [22.0, 61.0]	35.0 [0, 88.0]
	Freq (%)	Freq (%)	Freq (%)	Freq (%)
Missing	0 (0%)	0 (0%)	2 (40.0%)	2 (1.9%)
**Sex**				
Male	17 (40.5)	29 (50.0)	3 (60.0)	49 (46.7)
Female	24 (57.1)	29 (50.0)	0 (0)	53 (50.5)
Missing	1 (2.4)	0 (0)	2 (40.0)	3 (2.9)
**Marital status**				
Married	21 (50.0)	36 (62.1)	1 (20.0)	58 (55.2)
Single	17 (40.5)	11 (19.0)	2 (40.0)	30 (28.6)
Separated	2 (4.8)	2 (3.4)	0 (0)	4 (3.9)
Widowed	1 (2.4)	2 (3.4)	0 (0)	3 (2.9)
Missing	1 (2.4)	7 (12.1)	2 (40.0)	10 (9.5)
**Education level**				
Tertiary	9 (21.4)	12 (20.7)	3 (60.0)	24 (22.9)
None	1 (2.4)	5 (8.6)	0 (0)	6 (5.7)
Primary	3 (7.1)	4 (6.9)	0 (0)	7 (6.7)
Secondary	24 (57.1)	11 (19.0)	0 (0)	35 (33.3)
Missing	5 (11.9)	26 (44.8)	2 (40.0)	33 (31.4)
**Comorbidity**				
No	32 (76.2)	33 (56.9)	4 (80.0)	69 (65.7)
Yes	10 (23.8)	25 (43.1)	1 (20.0)	36 (34.3)

### Composition and relative abundance of taxa

**Fig 1:**
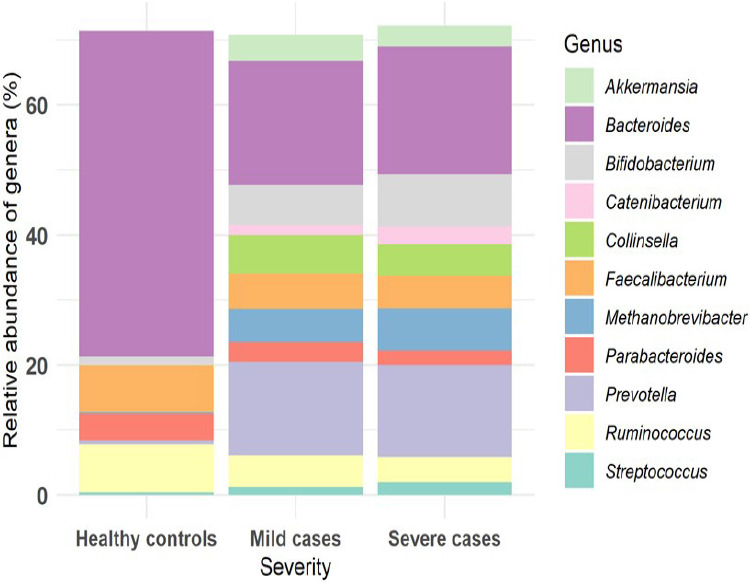
Relative abundance of genera

High-throughput sequencing of the v3-v4 variable region of the 16S rRNA gene generated a total of 5,658,826 sequence reads from the 105 stool samples. Quality assessment of the raw sequences was conducted using FastQC and none of the sequences were flagged as being of poor quality. The sequence lengths in the dataset were between 25 base pairs and 253 base pairs. Following taxonomic quantification, OTUs with very low abundances were filtered out; post-filtering, a significant proportion of the data was retained. The retained high-quality sequences yielded 5,180 OTUs, 12 phyla and 233 genera. The predominant phyla observed were Firmicutes, Fusobacteria, Bacteroidetes, Proteobacteria and Actinobacteria. The composition of the gut microbiome exhibited variability across the three categories: mild, severe, and healthy controls. At genus level, distinctive patterns were observed across the three groups. The genus Bacteroides was the most abundant across all groups, however there was a significant reduction among cases compared to healthy individuals. There was an increase of the Prevotella, Streptococcus, Akkermansia and Bifidobacterium genera in cases compared to healthy controls. Similarly, both Methanobrevibacter and Collinsella were highly abundant in both mild and severe groups while they were less abundant in the healthy controls. Parabacteroides and Ruminococcus appeared among the most abundant in all groups, but were more highly abundant in the healthy controls, Fig. 1.

**Fig 2:**
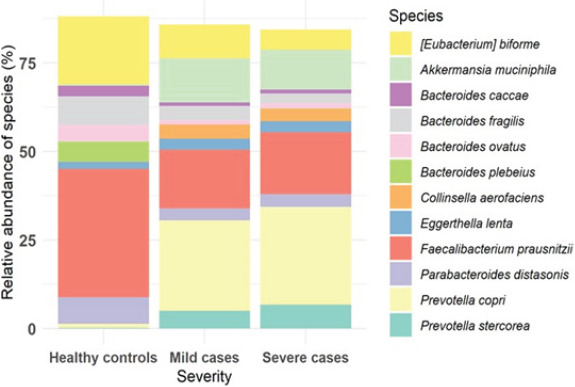
Relative abundance of species

Faecalibacterium prausnitzii was found in both mild and severe cases, but it was most abundant among healthy controls, followed by Eubacterium biforme. A few common species, such as Eggerthella lenta, Collinsella aerofaciens, and Parabacteroides distasonis, were abundant across the three groups. However, there was an over representation of some species in the mild cases and severe cases, with an increase in severe cases compared to mild cases. These species included Akkermansia muciniphila, Prevotella copri and Prevotella stercorea. Conversely, species like Bacteroides plebeius and Eggerthella lenta were more abundant in the healthy individuals, Fig 2.

### Pathogenic species

**Fig 3:**
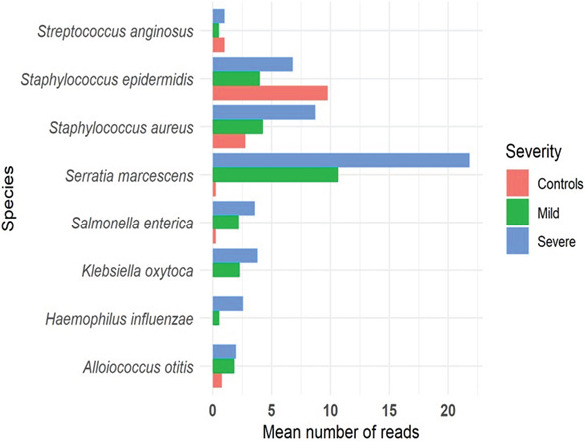
Relative abundance of pathogenic bacteria

There was a noticeable change in the relative abundance of species associated with disease states. Bacteria such as Haemophilus influenzae, Klebsiella oxytoca, and Serratia marcescens had an increased presence in the disease states, especially severe COVID-19 cases. Staphylococcus aureus and Staphylococcus epidermidis were both present across the three categories. However, for S. aureus, the abundance was highest in severe cases followed by mild cases. Salmonella enterica was almost negligible in controls, with a gradual increase in abundance from mild to severe cases. Klebsiella oxytoca was absent in the healthy group and had an increasing abundance from mild to severe, Fig 3.

Differential abundance analysis: Differential abundance analysis was done using R package DESeq2, to identify significant alterations in the microbial composition across the of COVID-19 groups. Taking mild cases as the reference, results showed taxa that significantly differ in abundance. Alphaproteobacteria, Anaerostipes, Chromatiales, Gracilibacteraceae, Peptococcaceae, and Thermoleophilia showed a significant decrease in their abundance, with negative log2 fold changes. Among them, Anaerostipes displayed the most pronounced decrease with a log2 fold change of -1.69985. Conversely, taxa including Bacillaceae, Bacillales, Bacilli, Enterococcaceae, and Enterococcus were significantly higher in abundance in severe cases. The Enterococcaceae family and the Enterococcus genus showcased the steepest increases with log2 fold changes of 2.817143 and 2.869012, respectively, Table 2.

**Table 2:** Differential abundance of taxa in mild vs. severe COVID-19 cases

	baseMean	log2FoldChange	lfcSE	stat	p value	padj
*Alphaproteobacteria*	4.101	-1.58	0.398	-3.97	0.016	0.007
*Anaerostipes*	1.431	-1.7	0.381	-4.463	0.005	0.002
*Bacillaceae*	5.875	1.924	0.473	4.069	0.016	0.007
*Bacillales*	10.347	1.732	0.405	4.272	0.01	0.004
*Bacilli*	180.808	1.493	0.378	3.952	0.016	0.007
*Chromatiales*	0.135	-1.402	0.356	-3.94	0.016	0.007
*Enterococcaceae*	7.315	2.817	0.525	5.368	0.000	0.000
*Enterococcus*	7.006	2.869	0.551	5.205	0.000	0.000
*Gracilibacteraceae*	0.502	-1.959	0.497	-3.942	0.016	0.007
*Peptococcaceae*	3.405	-1.239	0.294	-4.217	0.01	0.004
*Thermoleophilia*	0.06	-1.618	0.412	-3.924	0.016	0.007

**Fig 4:**
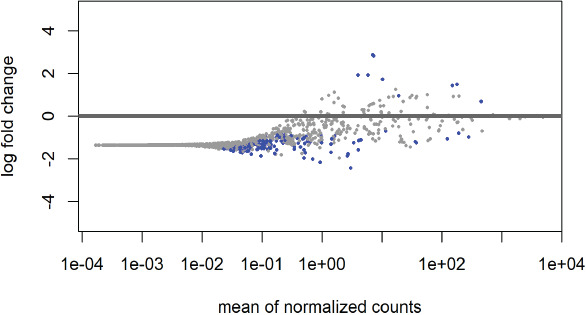
Differential abundance of taxa in mild vs. severe COVID-19 cases

A graphical representation of the differential abundance is shown in Fig 4 where the points are the different taxa. Blue points represent significant taxa while grey points represent non-significant taxa. Below the line are taxa that decreased among severe cases compared to mild cases.

### Diversity of the microbiome

Alpha diversity: Investigation of alpha diversity was done using the Shannon diversity index, between the mild and severe cases only, since health control groups had only a few samples. The findings suggest comparable microbial diversity between the two groups. Visual inspection of the plots showed no observable differences between the mild and severe cases (See supplementary Fig S1). Further, a statistical analysis using the Wilcoxon rank sum test was consistent with these observations. There was no significant difference in diversity between the two groups (p = 0.223)

**S1:**
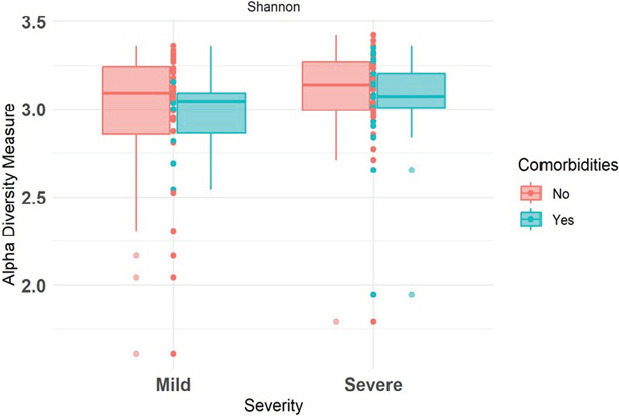
Alpha diveristy between mild and severe cases

Beta Diversity: Beta diversity comparison was done using the Bray-Curtis dissimilarity, and Principal Coordinates Analysis (PCoA) was employed to visualize the dissimilarities. The resulting PCoA plot showed three distinct clusters; however, both ‘mild’ and ‘severe’ COVID-19 cases were spread across all three clusters (see supplementary Fig S2). While there was a statistically significant difference in microbial community composition between different severity levels (p=0.013), the severity only explained about 3% of the variance in community composition.

**S2:**
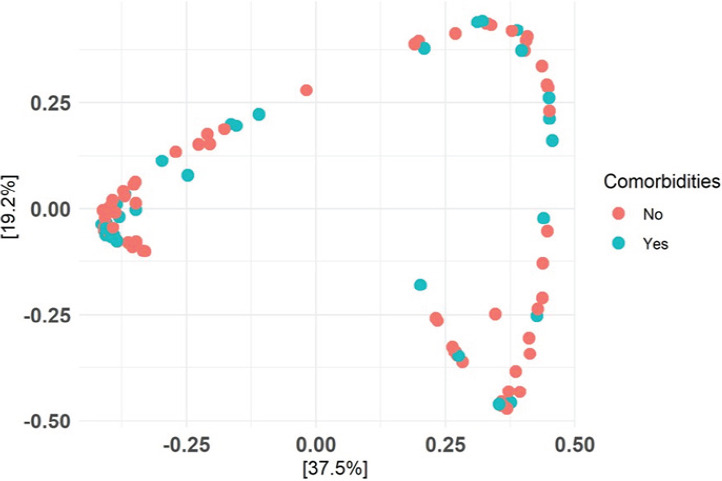
Beta diveristy between mild and severe cases

### Machine learning prediction of COVID-19 severity

We predicted severity of COVID-19 cases using diverse data points including the demographic characteristics and microbial composition of each sample. Requiring hospitalization was used a proxy for categorizing cases as severe or mild. We included sex, age and presence or absence of comorbidities as the other predictors. A range of models were explored including Logistic Regression which achieved an accuracy of 60.0%, Gradient Boosting which achieved 70.0%, Neural Networks which achieved 70.0%, and the Random Forests model, which was the top performer with an accuracy of 83.3%. Area under curve metrics for the each of the models are shown in supplementary figure Fig S3.

**S3:**
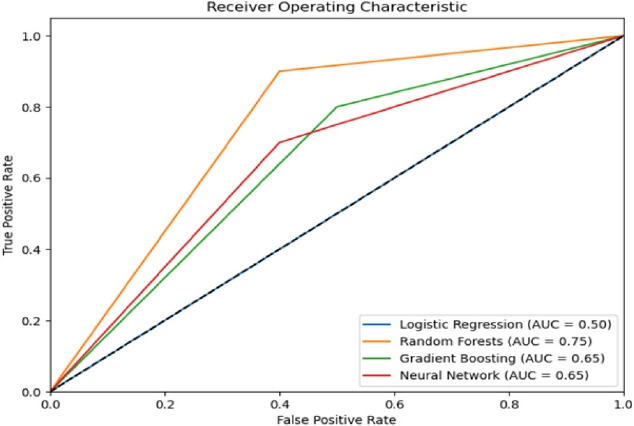
Area under the curve metrics for machine learning models

Utilizing predictions from the random forests model, we extracted feature importance metrics and ranked the most important features (taxa and factors) in the prediction of disease severity. Alongside age, several microbial taxa emerged as influential predictors, including Ruminococcaceae, Bacilli, Enterobacteriales, Porphyromonadaceae, Erysipelotrichales, Bifidobacteriaceae, and Prevotellaceae.

## Discussion

The composition of the gut microbiome in Uganda showed variability among three categories: mild COVID-19, severe COVID-19, and healthy individuals. A few common species, such as Eggerthella lenta, and Parabacteroides distasonis, were present among the most abundant species across all groups, signifying their abundance in the gut ecosystem. Conversely, some species including Prevotella copri and Akkermansia muciniphila (see Figure2) showed a significant increase in mild cases and an even higher increase in severe cases. This suggests that their abundance may be influenced by the health status of the individuals.

At the genus level, distinctive patterns were observed across the three groups. The increased presence of Prevotella, Akkermansia, Methanobrevibacter, and Collinsella (as shown in Figure 1) may indicate shifts related to the disease state. While some microbes were common across the groups, the variations in their abundance provide valuable insights into the microbial dynamics occurring in the gut. Similar studies in other populations have documented changes in gut microbiota composition during diseased states^1^,^2^,^29^-^31^. Understanding the increase or decrease in specific microbes could offer clues about their roles in health and disease. Further investigations into their functions might reveal their significance in the context of COVID-19.

Decrease in abundance of some taxa such as Bacteroides (Figure1) in higher disease severity aligns with existing literature which has emphasized the protective role of microbial diversity against pathogenic challenges. Promoting a diverse microbiome through dietary and therapeutic interventions could potentially reduce the severity of diseases like COVID-19^32^,^33^. Other studies in other populations have observed correlations between microbial composition and viral replication efficiency^33^,^34^. Understanding which microbes facilitate or hinder viral replication can lead to targeted therapeutic approaches.

Enrichment of opportunistic pathogens, such as Enterococcus species, and depletion of beneficial microbes, like Peptococcaceae, was observed in mild and severe COVID-19 cases (Table 2). A review of previous research^35^ suggested similar findings, noting a decrease in beneficial gut microbes with anti-inflammatory properties in COVID-19 patients. The increase in opportunistic pathogens can lead to infection, which is consistent with reports that COVID-19 patients frequently experience secondary bacterial infections, which can worsen disease outcomes^36^-^38^.

Previous studies incorporated machine learning to predict disease severity based on microbial compositions^39^,^40^. Distinct microbial signatures were identified, with the abundance of specific taxa in COVID-19 patients and a particular abundance of Prevotella in severe cases. This was similar to findings from a recent study^41^ which highlighted a protective role of the Prevotella genus in the long-term recovery process while other investigations^42^,^43^ reported varied results. The variation could be attributed to various factors like sample collection methods, patient demographics, or even geographic variations in microbial populations. If Prevotella or other bacteria play a direct or indirect role in disease exacerbation, interventions targeting these bacteria might be explored as a treatment or preventive strategy.

## Limitations

There were certain limitations in our study; first is an imbalance between cases and controls i.e., the household healthy contacts whom we used as a comparator group. This resulted from lockdowns during the enrollment period that made it difficult to enroll healthy contacts due to restrictions in movement. Additionally, some factors like prior antibiotic usage and diet which might influence the microbiome, might have not been elaborately reported by participants.

## Conclusion

There is an association between specific taxa and COVID-19 severity in Ugandan cases however, more comprehensive and longitudinal studies are necessary to validate this.

## List of Abbreviations

COVID-19: Corona Virus Disease -2019
DNA: Deoxyribonucleic acid
PCR: Polymerase chain reaction
PERMANOVA: Permutational multivariate analysis of variance
RT-PCR: Real-time reverse transcriptase-polymerase chain reaction
SARS-CoV-2: severe acute respiratory syndrome coronavirus 2
